# Macrophage Involvement in Aging-Associated Skeletal Muscle Regeneration

**DOI:** 10.3390/cells12091214

**Published:** 2023-04-22

**Authors:** Chang-Yi Cui, Luigi Ferrucci, Myriam Gorospe

**Affiliations:** 1 Laboratory of Genetics and Genomics, National Institute on Aging Intramural Research Program, National Institutes of Health, Baltimore, MD 21224, USA; 2 Translational Gerontology Branch, National Institute on Aging Intramural Research Program, National Institutes of Health, Baltimore, MD 21224, USA

**Keywords:** skeletal muscle, macrophage, satellite cell, fibro-adipogenic progenitor, regeneration, repair, aging

## Abstract

The skeletal muscle is a dynamic organ composed of contractile muscle fibers, connective tissues, blood vessels and nerve endings. Its main function is to provide motility to the body, but it is also deeply involved in systemic metabolism and thermoregulation. The skeletal muscle frequently encounters microinjury or trauma, which is primarily repaired by the coordinated actions of muscle stem cells (satellite cells, SCs), fibro-adipogenic progenitors (FAPs), and multiple immune cells, particularly macrophages. During aging, however, the capacity of skeletal muscle to repair and regenerate declines, likely contributing to sarcopenia, an age-related condition defined as loss of muscle mass and function. Recent studies have shown that resident macrophages in skeletal muscle are highly heterogeneous, and their phenotypes shift during aging, which may exacerbate skeletal muscle deterioration and inefficient regeneration. In this review, we highlight recent insight into the heterogeneity and functional roles of macrophages in skeletal muscle regeneration, particularly as it declines with aging.

## 1. Introduction

The skeletal muscle comprises approximately 40% of body mass and 50–75% of body proteins in humans [1]. The primary function of the skeletal muscle is to support posture and movement, but it is also deeply involved in metabolism and thermoregulation [2,3,4]. The skeletal muscle experiences microinjuries or trauma arising from physical labor, exercise, accidents, and daily activity, and thus it undergoes continual repair and regeneration. During aging, however, damage accumulates and regenerative capacity declines, which can result in loss of muscle mass and function, a condition known as sarcopenia [5,6,7]. Skeletal muscle degeneration begins in humans in their 30s and accelerates with age; nearly 50% of people over 80 years of age have some degree of sarcopenia [8,9,10]. Aging-related skeletal muscle deterioration leads to frailty, confinement, falls and susceptibility to metabolic syndrome, severely affecting the quality of life of older persons.

Extrinsic and intrinsic injury in skeletal muscle resident cells and altered interactions between cell types during aging promote skeletal muscle deterioration. For example, substantial denervation takes place during aging due to atrophy of motoneurons [11]. Orphan myofibers are then preferentially reinnervated by type I motoneurons, causing a fiber type switch toward a prevalence of type I myofibers, and ultimately causing a decline in muscle function [11]. In addition, chronic unrepaired damage leads to myofiber apoptosis and atrophy [12]. Meanwhile, myogenic satellite cells (SCs) show reduced proliferative and differentiation capacity and the communication between myogenic and non-myogenic cells is impaired [13,14,15]. SCs and fibro-adipogenic progenitor cells (FAPs) are crucial for skeletal muscle repair and regeneration, but the regeneration process is largely orchestrated by polarized macrophages [16,17,18,19]. Understanding macrophage action is thus critical for understanding skeletal muscle regeneration and aging. In this review, we discuss skeletal muscle macrophages and their interactions with the major constituent cells of the skeletal muscle during regeneration, with a particular focus on aging.

## 2. Major Constituent Resident Cells and Their Involvement in Skeletal Muscle Aging

The skeletal muscle comprises terminally differentiated multinuclear muscle cells (myofibers), and populations of mononuclear cells including myogenic SCs and non-myogenic “accessory” cells, e.g., FAPs, fibroblasts, adipocytes, and innate and adaptive immune cells [20,21]. Loss of muscle mass and strength, increased infiltration of fat and fibrotic tissue, as well as deterioration of neuromuscular junctions (NMJs) and local circulation are predominant features of skeletal muscle aging [22,23,24]. Myogenic and non-myogenic stem/progenitor cells and innate immune cells play a critical role in skeletal muscle regeneration and aging. In this section, we discuss the involvement of SCs and FAPs in skeletal muscle aging; we will separately discuss the function of macrophages, the predominant innate immune cell population in skeletal muscle, in Section 3, Section 4, Section 5, Section 6, Section 7, Section 8 and Section 9 below.

### 2.1. SCs and Skeletal Muscle Aging

SCs are the sole myogenic adult stem cells in skeletal muscle [25,26] and they are located between the sarcolemma and the basal lamina in each myofiber [27]. Activated SCs proliferate and differentiate to become myoblasts, which then evolve into myocytes and fuse to form myotubes; myotubes in turn merge to form multinucleated myofibers. Three main types of myofibers are found in adult human skeletal muscle, oxidative slow-twitch (type I), glycolytic fast-twitch (type IIX), and intermediate oxidative-glycolytic fast-twitch (type IIA) myofibers, although it has been suggested that this classification is not clear cut [1,28]. Mice have an additional type of myofiber known as very fast-twitch (type IIB) myofibers [29].

During aging, changes intrinsic to SCs and changes in the SC microenvironment induce chronic SC activation and apoptosis, resulting in exhaustion of the SC pool [14,30]. The remaining SCs in old skeletal muscle show reduced capacity of activation, proliferation, and differentiation following injury [14,31]. SCs are heterogeneous [25,30] and can be found in deeply quiescent, quiescent or activated states [32,33]. Recent studies using single nuclei identified four subpopulations of SCs, including PAX7+ “quiescent”, MYOG+ “transient differentiating”, TNFRSF12A+ “activated”, and ICAM1+ “immune-responsive” subpopulations in adult human skeletal muscle [34]. All subpopulations were reduced in number during aging, but the “transiently differentiating” subpopulation showed the most striking decline, underscoring the fact that the initial phase of stem cell activation is compromised in muscle from aged individuals [34,35].

Approximately two-thirds of SCs from aged mouse skeletal muscle show intrinsic defects and cannot properly repair myofibers or repopulate the SC reservoir [36]. This dysfunction likely stems from increased function of p38 or p16, as inhibition of p38 signaling or silencing of p16 gene restored quiescence and regenerative function of old SCs [36,37]. Signaling through p38 and p16 induces cell senescence by inhibiting CDK4/6 [38], and thus senescence is an important contributor of deterioration of SC function during aging. A more recent study reported the accumulation of a subgroup of dysfunctional SCs that express high levels of the integrin-associated receptor protein CD47, which mediates a suppressive action of the THBS1 (Thrombospondin 1) ligand on SC proliferation in old mouse skeletal muscle [39]. Notably, CD47 is a typical signal to suppress phagocytosis, enabling cancer cells to escape from surveillance by macrophages [40,41,42]. Thus, compromised phagocytotic clearance due to elevated CD47 may contribute in part to the accumulation of non-proliferative SCs in old skeletal muscle.

The microenvironment is also critical for the maintenance of SC quiescence and self-renewal [13]. SCs were shown to produce collagen V (COLV), a critical structural component of the SC niche, in a Notch signaling-dependent way [43]. COLV interacts with SCs through the calcitonin receptor, and depletion of COLV induced SCs to enter the cell cycle and thereby reduced the SC pool in mice [43]. During aging, the extracellular matrix (ECM) in the skeletal muscle SC niche undergoes active remodeling [44]. The ECM protein SMOC2 (SPARC-Related Modular Calcium Binding 2), produced by FAPs, increased in the SC niche of aged mouse skeletal muscle, and impaired SC self-renewal [44]. Furthermore, the proinflammatory microenvironment of the SC niche elicited by the senescence-associated secretory phenotype (SASPs) from neighboring senescent cells blunted SC activation during injury repair [45]. A more recent study showed that old SCs can be reprogrammed back to the youthful state when transplanted to the SC niche in young mice, affirming the key role of the microenvironment on SC homeostasis and aging [30]. Collectively, both intrinsic and extrinsic changes contribute to the decline of SC number and function during skeletal muscle aging.

Notably, the aging-related decline of muscle mass does not happen uniformly in all muscle fibers. Type I myofiber is known as “aging resistant”, while type II myofiber is known as “aging prone” [34,46,47]. It remains to be determined whether distinct SC subpopulations reside in different types of myofibers and if repair and regeneration takes place in a myofiber-dependent manner during aging.

### 2.2. FAPs and Skeletal Muscle Aging

All non-myogenic cells, including FAPs, are localized in intra- and intermuscular connective tissues [16,17]. FAPs are mesenchymal stromal progenitor cells that have the potential to differentiate into fibroblasts or adipocytes in skeletal muscle [48]. In the steady state, FAPs provide supportive microenvironment for long-term homeostatic maintenance of SCs, in part by producing ECM proteins [44,49,50]. FAPs also secrete cytokines, including IL6, GDF10, IGF-1, and WISP1, remove cell debris by phagocytosis, and interact with SCs and innate immune cells during skeletal muscle injury repair and regeneration [49,51]. FAPs are activated by eosinophils upon injury and promote SC activation [51,52]. At late repair stages, FAPs are activated by M2 macrophages and produce ECM components that help complete the repair [53].

FAPs are a heterogeneous population [30,54]. In the homeostatic state, most FAPs comprise a large Tie2^low^ subgroup and a small Tie2^high^ subgroup, which correlated with neo-angiogenesis and muscle growth, respectively [54]. During injury repair or in Duchenne Muscular Dystrophy (DMD) models, a transient third subgroup of FAPs emerges, Vcam1^high^, that shows profibrotic properties [54]. During aging, FAPs number and function decline [50]. A recent study identified an increase in a p16+ FAP subpopulation in old skeletal muscle in mice and humans [20], suggesting that senescent FAPs accumulate during aging. Aged FAPs secrete less WISP1 (WNT1-inducible signaling pathway protein 1), a critical activator of SCs, resulting in impaired muscle repair and regeneration in mice [50]. Furthermore, bidirectional FAPs skew to fibrogenic fate during aging, leading to increased deposit of ECM proteins in old skeletal muscle toward stiffness or fibrosis during injury repair [50]. Changes in FAPs during aging thus impair skeletal muscle repair and regeneration.

It is worth noting that we and others found that collagens were unchanged or even increased at the protein level, but decreased markedly at the mRNA level in old skeletal muscle in mice [55,56,57] and humans [34,35]. This discrepancy can be explained in part by decreased ECM protein turnover, elevated ECM crosslinking, reduced ECM degradation, or increased synthesis of ECM proteins promoted by macrophages during normal aging [55,56,57,58], although the exact mechanisms remain to be elucidated.

During aging, adipocytes have been found to fill the intrafibrillar spaces left by degenerated myofibers [59,60]. The rise in intra- and intermuscular fat in skeletal muscle with aging has been associated with sarcopenia, aberrant metabolism, and chronic inflammation [61,62]. Proinflammatory cytokines inhibit, while anti-inflammatory cytokines promote adipogenesis by FAPs in culture [63,64]; hence, the overall rise in systemic inflammation with aging does not explain the fat increase in old skeletal muscle. Given that FAPs tend to be fibrogenic during aging [50], FAPs may not be the sole contributors to the rise in fat in aged skeletal muscle. The mechanism of fat increase in skeletal muscle during aging remains unclear, but a systemic decrease in sex hormones, leptin sensitivity, lack of exercise, and/or reduced mitochondrial function with reduced utilization of lipids for oxidative phosphorylation may contribute to the heightened fat levels in aged skeletal muscle [59].

## 3. Introduction to Macrophages

Macrophages are a group of highly heterogeneous innate immune cells that provide a first line of defense, but also present antigens to adaptive immune cells, induce or resolve inflammation, remove dead cells or cells debris, and repair and remodel tissues [65,66,67,68]. In this section, we first provide general information about the origin, polarization, function, and tissue-specific actions of macrophages; afterwards, we focus on skeletal muscle macrophages.

### 3.1. Origin of Macrophages

Discovered as professional phagocytes by Metchnikov more than a century ago [69], macrophages can be divided into tissue-resident and non-resident macrophages [70]. Tissue-resident macrophages include embryo-derived, self-renewing macrophages, and adult bone marrow-derived, non-renewing macrophages. Typical self-renewing macrophages derived from the embryonic yolk sac or fetal liver include microglia of the brain, Kupffer cells of the liver, alveolar macrophages of the lung, and Langerhans cells of the skin [70]. Non-renewing resident macrophages in the intestine, pancreas, and skin dermis are continuously replenished in each tissue by circulating monocytes derived from the bone marrow [71]. Recent studies have shown that many tissues, including skeletal muscle, pancreas, and adipose tissue, have mixed populations of self-renewing and non-renewing resident macrophages [72,73,74,75]. Non-resident macrophages are derived from blood monocytes and infiltrate in tissues following injury or infection [76]. It remains unclear whether macrophages with distinct origins behave differently in vivo.

### 3.2. Macrophage Polarization, Heterogeneity, and Function

Macrophages are highly versatile cells. The functional diversity of macrophages largely arises from their ability to polarize, a complex and dynamic process that enables them to differentiate into distinct subgroups and perform specialized functions [77]. For example, depending on signals from the local milieu or cell-intrinsic conditions, macrophages can polarize into classical M1 or alternative M2 subtypes. Triggers such as interferon γ (IFNγ), lipopolysaccharides (LPS), and granulocyte-macrophage colony-stimulating factor (GM-CSF) can direct macrophage polarization to M1 status, while interleukin (IL) 4, IL13, or glucocorticoids promote M2 polarization [78,79]. Polarized M1 macrophages acquire pro-inflammatory properties with host defense function partly through upregulation of the nitric oxide synthase (iNOS) pathway. By contrast, M2 macrophages gain anti-inflammatory character and tissue repair ability, in part by upregulating the arginase pathway [80,81]. The dichotomy of M1 and M2 has been helpful to explain some of the strikingly different actions of macrophages, but they appear to represent two distant groups in a multidimensional polarization landscape [77,81,82,83].

In this regard, recent single-cell-based transcriptomic and flow cytometric analyses have identified additional macrophage subgroups in several tissues, including two in lung interstitium, three in artery, and three in adipose tissue of obese mice [71,75,84,85,86]. A more recent study has shown that three distinct macrophage subgroups were distributed across tissues including heart, liver, kidney, lung, and brain [75]. However, the proportion of each subgroup in different tissues was variable, consistent with the tissue-dependent heterogeneous functions of macrophages [75]. Collectively, these macrophage subgroups had some similarities, but also some distinctions from traditional M1 or M2 macrophages [22].

### 3.3. Mechanism of Tissue-Specific Actions of Macrophages

In addition to general functions, tissue-resident macrophages also display tissue-specific functions. For example, microglia regulate synaptic pruning [87], adipose tissue macrophages are involved in thermoregulation and lipolysis [88], and cardiac macrophages facilitate electrical conduction [89]. In agreement with this functional diversity, tissue-resident macrophages display tissue-dependent transcription patterns, which are characterized by specific transcriptomic programs in each tissue [65,90]. Microglia development was determined by transcription factors SMADs and SALL1 activated by the TGFβ pathway, while peritoneal macrophages were specified by GATA6 downstream of retinoic acid [91,92,93]. Such tissue-dependent patterns of transcription by macrophages can be determined at least in part by specific tissue microenvironments, which promote the formation of a tissue-specific enhancer landscape and thus establish tissue-dependent gene expression patterns and identities [90,92]. In support of this notion, peritoneal macrophages transplanted to the lung were shown to lose their original expression pattern and adopted a lung macrophage transcriptome [92]. These studies underscore the fact that macrophages adapt to local tissue environments and acquire tissue-specific functional identities.

## 4. Origin of Skeletal-Muscle-Resident Macrophages

Macrophages are a predominant population of immune cells in skeletal muscle, and they localize in the perimysium and endomysium areas [56]. In our studies, ~2–5% of mononuclear cells from steady-state mouse skeletal muscle were CD45+/CD11b+/F4/80+ macrophages [94,95].

Resident macrophages in adult skeletal muscle were originated from both embryonic hematopoietic progenitors and adult bone marrow hematopoietic stem cells, as shown by analysis of lineage tracing and bone marrow transplantation [72]. Almost all skeletal muscle macrophages were derived from the embryonic yolk sac or liver at early embryonic stages. However, bone marrow-derived CCR2+ monocytes were recruited to skeletal muscle from late embryonic stages and increased in number continuously during postnatal stages [72]. At 3 to 6 months of age, ~50–60% of skeletal muscle macrophages in mice were found to be derived from bone marrow hematopoietic stem cells, indicating that skeletal muscle macrophages comprise mixed origins in the adult.

Furthermore, almost all M1-like macrophages in adult skeletal muscle were derived from bone marrow hematopoietic stem cells, while M2-like macrophages were originated from both embryonic hematopoietic progenitors and adult bone marrow stem cells [72]. Notably, a small subcluster of proliferating skeletal muscle macrophages was identified by two independent groups using single-cell transcriptomic analysis [72,94]. This subpopulation expressed high levels of mRNAs encoding cell cycle-related proteins, including *Mki67*, *Cdk1*, and *Top2a* mRNAs, and it may represent embryo-derived self-renewing macrophages in mouse skeletal muscle. The origin of human skeletal muscle macrophages, however, remains to be elucidated.

## 5. Skeletal-Muscle-Resident Macrophages: Polarization and Heterogeneity

To perform the diverse functional needs of the skeletal muscle, macrophages undergo massive polarization that results in a high degree of heterogeneity. Traditionally, the polarization or heterogeneity of skeletal muscle macrophages was defined by membrane markers. In recent years, however, single-cell transcriptomic studies have allowed this classification to gain higher resolution. Below we describe an updated understanding of skeletal muscle macrophage classification using membrane markers and single-cell transcriptomic analysis.

### 5.1. Membrane Marker-Based Classification

Membrane markers are effective for identifying macrophage subgroups in tissues, and enable their isolation for further studies. Membrane proteins including CD11b and F4/80 have been widely used as traditional markers of macrophage-enriched immune cell population in human and/or mouse skeletal muscle [55,56,83]. The pattern recognition receptor CD206 was the most widely used membrane marker of M2 macrophages, while CD86, CD80 and MHCII proteins were used to identify M1 macrophages [96,97]. Based on the presence of these markers, M1 and M2 macrophages were identified from steady-state skeletal muscle from humans and mice by immunohistological approaches [55,56,83]. However, recent studies suggest that those traditional markers may not be ideal to define macrophage polarization in skeletal muscle. Flow cytometric analysis revealed that most macrophages isolated from human skeletal muscle simultaneously expressed the M2 marker CD206 and the M1 marker CD86 [83]. It was also found that CD206 and CD86 were expressed in ~80% of macrophages isolated from mouse skeletal muscle and most macrophages simultaneously expressed the mRNAs encoding these markers by single-cell RNA-sequencing (scRNA-seq) analysis [94].

**Figure 1:**
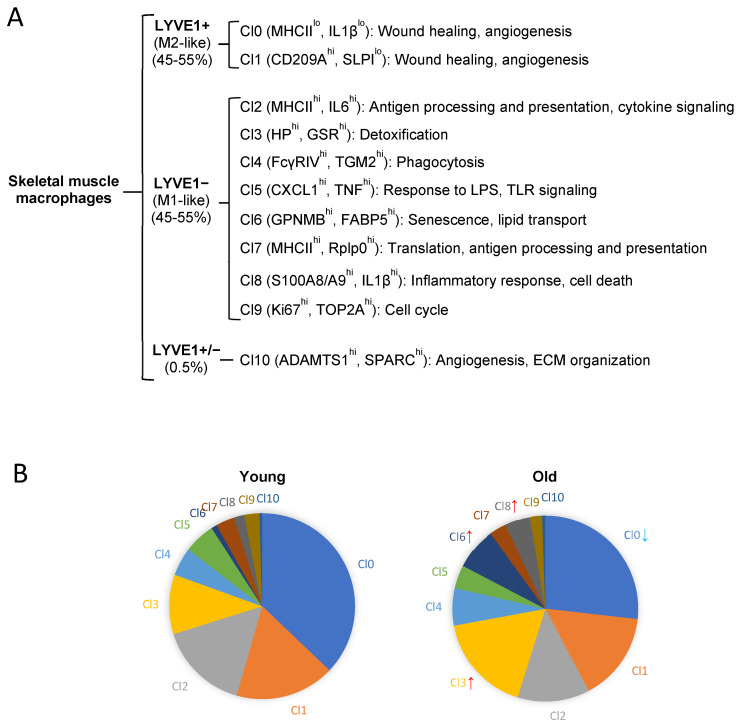
Macrophage heterogeneity in mouse skeletal muscle and its changes with aging. (**A**) Mouse skeletal muscle macrophages can be divided into two large groups, LYVE1+ (M2-like) and LYVE1− (M1-like). LYVE1+ macrophages comprise two clusters, both of which show reparative function. LYVE1− macrophages comprise eight clusters with various functions. An additional small cluster (Cl10) includes both LYVE1+ and LYVE1− macrophages. Cl10 macrophages exclusively express ADAMTS1, which was shown to activate SCs. (**B**) Polarization shifts during aging. The size of Cl0 decreased (blue arrow), while Cl3, Cl6 and Cl8 increased (red arrows) in old mouse skeletal muscle.

In an effort to identify more informative membrane markers, we found that another membrane protein, LYVE1 (lymphatic vessel endothelial hyaluronan receptor 1), almost evenly divided CD45+/CD11b+/F4/80+ macrophages isolated from mouse skeletal muscle into two groups by scRNA-seq and flow cytometric analyses [94] (Figure 1A). LYVE1 was recently used to classify macrophages in lung interstitium, heart, liver, kidney, and brain in mice [71,75,85]. LYVE1+ macrophages displayed M2-like properties, with increased expression of mRNAs encoding proteins involved in angiogenesis and tissue repair [94]. In contrast, LYVE1− macrophages expressed elevated levels of mRNAs encoding pro-inflammatory and antigen-processing and -presentation proteins [94]. Notably, >99% of LYVE1+ macrophages expressed *Cd206* mRNA, while ~60% of LYVE1− macrophages also expressed *Cd206* mRNA, although the expression levels were lower in LYVE1− macrophages in scRNA-seq analysis [94]. Based on these observations, we proposed that LYVE1 may be a better membrane marker to define M2-like and non-M2-like (M1-like) macrophages in mouse skeletal muscle [94]. LYVE1^high^ and LYVE1^low^ macrophages have been identified in human lungs, and they expressed slightly distinct transcriptomes from mouse lung macrophages [71]. The expression pattern of LYVE1 in human skeletal muscle macrophages remains to be analyzed.

By adding an M1 membrane marker, MHCII, we identified four distinct subgroups of mouse skeletal muscle, including M2-like (LYVE1+/MHCII^low^), M1-like (LYVE1−/MHCII^high^), M1-M2 intermediate (LYVE1+/MHCII^high^), and a novel subgroup of LYVE1−/MHCII^low^, by flow cytometric and scRNA-seq analyses [94]. The M1-M2 intermediate subgroup displayed both M1- and M2-like traits. This subgroup may have the potential to shift to more differentiated M1- or M2-like subgroups depending on the surrounding microenvironment. The novel LYVE1−/MHCII^low^ subgroup was clearly separated from the other three subgroups by flow cytometric analysis [94]. Notably, however, ~one-half of macrophages from this subgroup exhibited a remarkably robust phagocytic capacity; this group, characterized as being FcγRIV+/CD36+, comprised macrophages that were deemed “super-phagocytic” in an earlier cancer study [98]. The function of this subgroup in skeletal muscle warrants further study (see Section 5.2 and Section 9.1 below).

### 5.2. Single-Cell Transcriptomic-Based Unsupervised Classification

Specific patterns of gene expression determine the identity and function of a cell or cell group. Accordingly, analysis of the transcriptome by scRNA-seq analysis may enable the identification of functional traits of macrophage subgroups in skeletal muscle. Unsupervised clustering of scRNA-seq data identified 11 macrophage clusters from young and old mouse skeletal muscle [94] (Figure 1A). LYVE1+ M2-like macrophages comprised only two clusters, and both clusters showed similar reparative function [94]. By contrast, LYVE1−, M1-like macrophages comprised 8 distinct functional clusters, suggesting that M1-like macrophages are more heterogenous than M2-like macrophages in mouse skeletal muscle. These differences may also explain the current absence of a universal marker to identify all M1-like macrophages, unlike M2-like macrophages, which can be easily recognized by markers LYVE1, FOLR2, or CD206 [83,94].

Distinct clusters in LYVE1−, M1-like macrophages differentially express mRNAs encoding proteins MHCII, IL6, IL1β, TNF, CXCL1, FCγRIV, GPNMB, FABP5, KI67, and S100A8/A9 proteins. The clusters in M1-like macrophages correlated with functions in antigen processing and presentation, inflammation, cellular detoxification, phagocytosis, lipid transport, senescence, protein synthesis, and proliferation (Figure 1A).

Single-cell transcriptomic studies identified several novel functional macrophage subgroups in mice. For example, a GPNMB+ cluster (Cluster 6, Cl6, in Figure 1A) was enriched in mRNAs encoding senescence-related proteins including GPNMB, SPP1, CTSD and GDF15 [99,100,101,102,103]. Notably, a group of mRNAs encoding lipid transport and metabolism-related proteins, including FABP5, FABP4, APOE, TREM2, PLIN2, CD68 and LPL [104,105,106,107,108,109], were also enriched in this cluster. GPNMB was highly expressed in senescent cells in vivo, and GPNMB vaccination or genetic ablation of *Gpnmb* attenuated aging-related phenotypes in mice [101]. GBNMB was exclusively expressed in this cluster [94].

S100A8+/A9+ cluster (Cl8) expressed high levels of mRNAs encoding proinflammatory proteins S100A8, S100A9, THBS1, RETNLG, and IL1β [94]. S100A8 and S100A9, two protein biomarkers of inflammatory diseases [110], were exclusively expressed in this cluster. Together with GPNMB+ macrophages, this cluster may contribute to proinflammatory and senescence traits in old mouse skeletal muscle.

A unique FcγRIV+/CD36+ cluster (Cl4) was identified that expressed high levels of mRNAs encoding phagocytotic proteins FcγRIV, TGM2, MYO1G, PPARγ, and CD36 [94]. This cluster was a core component of the “super-phagocytic” LYVE1−/MHCII^low^ macrophages [94]. Interestingly, this cluster expressed high levels of the mRNA encoding CD47, a transmembrane glycoprotein having dual functions as ligand and receptor [111]. CD47 is a typical “don’t eat me” signal, which prevents cells from clearance by phagocytes [40,41,42]. Thus, while displaying strong phagocytic capability, macrophages in this cluster protect themselves from other phagocytes by expressing CD47.

The smallest cluster (Cl10) was a mixed population of LYVE1+ and LYVE1− macrophages (Figure 1A). *Adamts1* mRNA, encoding ADAMTS1 (a disintegrin and metalloproteinase with thrombospondin motif 1), was exclusively expressed in this cluster. Macrophage-derived ADAMTS1 was shown to activate SCs at early muscle injury repair stages by suppressing NOTCH1 signaling and thus promoted muscle repair and regeneration in mice [112].

In sum, membrane marker-based and single-cell transcriptomics-based classifications have uncovered great heterogeneity in mouse skeletal muscle macrophages in the homeostatic state. Notably, the heterogeneity seen in skeletal muscle macrophages was distinct from that of resident macrophages in other tissues in mice, as shown in a recent study [72], consistent with the tissue-dependent gene expression patterns and functions of macrophages. Compared to the membrane marker-based classification, clusters identified from unsupervised clustering generally lacked specific membrane markers for conventional identification, but likely define macrophage polarization more precisely. Macrophage clusters in human skeletal muscle remain to be characterized.

## 6. Function of Skeletal Muscle Macrophages in Repair and Regeneration

**Figure 2:**
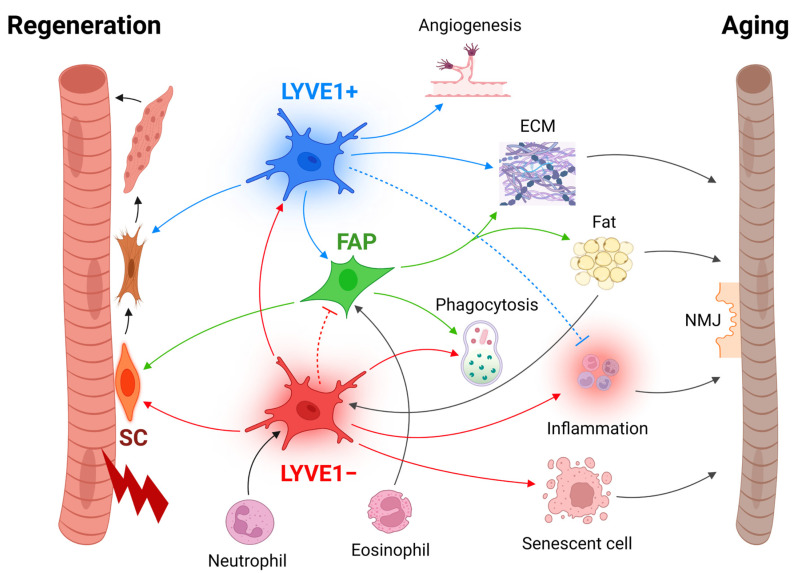
Skeletal muscle macrophages orchestrate injury repair and regeneration. Upon injury, macrophages polarize to proinflammatory M1-like (LYVE1−, red) status primarily by neutrophils. M1-like macrophages activate SCs to proliferate, while they induce inflammation and clean up dead cells by phagocytosis. Meanwhile, eosinophils activate FAPs (green), which further activate SCs to proliferate and remove cell debris by phagocytosis. M1-like macrophages can induce apoptosis in FAPs to prevent excessive fibrogenesis. At late repair stages, M1-like macrophages shift to M2-like macrophages (LYVE1+, blue). M2-like macrophages promote myoblast differentiation to form myotubes and further myofibers. M2-like macrophages suppress inflammation and facilitate angiogenesis and fibrogenesis toward completion of the repair directly and through FAPs. During normal aging, skeletal muscle macrophages generally shift to a proinflammatory state, and some of them may undergo senescence. Increased fat in skeletal muscle during aging may promote these changes. Along with increased fibrogenesis (ECM), changes in macrophages may promote NMJ deterioration and skeletal muscle aging.

Macrophages have critical roles in the skeletal muscle, probably best illustrated by the process of muscle injury repair and regeneration, during which they mediate inflammation, dead cell removal, and tissue repair and remodeling by shifting polarization states [18,19]. Upon injury, damage-associated molecular patterns (DAMPs) from broken myofibers recruit innate immune cells including neutrophils, eosinophils, and monocytes to the injury site [18,19,51,53,113]. As neutrophils fight off pathogens and remove cell debris, they also promote M1 polarization of recruited monocytes by secreting proinflammatory cytokines at early stages (Figure 2). Polarized M1 macrophages then largely take over the role of neutrophils, and further activate SC proliferation by secreting proinflammatory cytokines and growth factors, including IFNγ, TNF, HGF, PGE2, and ADAMTS1 [18,19,112,114]. Macrophages interact with SCs not only via paracrine factors, but also directly by physical cell-to-cell interactions [115,116]. In addition, FAPs activated by eosinophils further promote SC proliferation and necrotic debris removal [51,52].

At late repair stages, M1 macrophages transition to M2 macrophages thanks to anti-inflammatory cytokines secreted by regulatory T lymphocytes (Treg cells) and by phagocytosed cell debris [18,117,118]. The transitioned M2 macrophages along with resident M2 macrophages suppress inflammation, promote SC-derived myoblast differentiation, promote angiogenesis, and activate FAPs to produce ECM to complete the repair and regeneration (Figure 2) [19,22,53,119,120,121]. The excess of recruited macrophages is likely cleared by local apoptosis rather than emigrate to lymph nodes at the last repair stage [122,123,124]. Fine-tuning of the macrophage function is evidenced in the macrophage-FAP interaction during repair and regeneration. M1 macrophages are found to induce FAP apoptosis by secreting TNF during early repair stages to clear excessive FAPs and thus prevent fibrosis [121]. By contrast, M2 macrophages can suppress promyogenic action but promote profibrogenic function of FAPs [125]. Thus, excessive M2 macrophages impair skeletal muscle regeneration [125].

Supporting the important function of macrophages in muscle repair and regeneration, systemic depletion of macrophages resulted in delayed injury repair [117,126,127]. Consistently, macrophages were indispensable for injury repair in engineered muscle tissues in vitro and in vivo [128]. Furthermore, injection of IL4-conjugated gold nanoparticles into ischemia-induced muscle injury site increased M2 macrophages and reduced M1 macrophages during late repair stages, which improved skeletal muscle repair and regeneration in mice [129]. Thus, the collective evidence strongly supports a main role for macrophages in orchestrating skeletal muscle repair and regeneration by interacting with SCs and FAPs.

## 7. Shifts in Skeletal Muscle Macrophage Polarization during Aging

Macrophage polarization shifts during aging, likely the result of aging-related systemic or local microenvironmental changes, or cell-intrinsic changes like mitochondria dysfunction, autophagy impairment, or endoplasmic reticulum (ER) stress [81]. We will discuss phenotypic changes in macrophages during skeletal muscle aging in this section, and macrophage involvement in skeletal muscle function in Section 8 below.

Both scRNA-seq and flow cytometric analyses revealed that LYVE1+ M2-like reparative macrophages decreased, and LYVE1− M1-like proinflammatory macrophages increased in old mouse skeletal muscle [94]. Accordingly, the expression levels of M2 markers (*Lyve1* and *Folr2* mRNAs) declined, while proinflammatory and senescence-related markers (*S100a8*, *S100a9*, *Il1β*, *Spp1* and *Gpnmb* mRNAs) increased in old skeletal muscle macrophages [94], suggesting an overall phenotypic shift towards a proinflammatory state with aging. However, earlier studies using immunohistology and flow cytometry showed that CD206+ M2-like anti-inflammatory macrophages increased or remained unchanged in old skeletal muscle [55,56,130,131]. Thus, different membrane markers and analytical methods provided different results. Technical limitations to consider include the smaller numbers of macrophages that can be analyzed by immunohistology, as well as the possible bias that flow cytometry or scRNA-seq analysis can introduce during the isolation of muscle macrophages. Thus, more specific markers and better detection methods are required for a more accurate evaluation of polarization shifts during aging. At present, LYVE1 may be a better marker of M2-like macrophages, especially in mouse skeletal muscle, and LYVE1-based classification suggested a proinflammatory shift during skeletal muscle aging.

Notably, unsupervised classification suggested that the phenotypic shift most likely stemmed from changes in a few specific clusters during aging (Figure 1B). GPNMB+ cluster (Cl6), expressing mRNAs that encode senescence-associated and lipid transport proteins, was barely detectable in young skeletal muscle, but greatly expanded in the old (Figure 1B) [94]. Lipid transporter genes were found to be elevated and lipid droplets were increased in the cytoplasm of senescent skeletal muscle macrophages [45]. A rise in lipids promotes inflammation and insulin resistance, largely mediated by macrophages [132]. Increased fat infiltration during skeletal muscle aging may promote senescence in GPNMB+ macrophages and further contribute to the proinflammatory conversion in old skeletal muscle in mice. The S100A8+/S100A9+ cluster (Cl8), expressing high levels of proinflammatory genes, was also significantly increased in old skeletal muscle, while a reparative cluster (Cl0) declined during aging (Figure 1B) [94]. Thus, only a few clusters shifted in phenotypes during aging, suggesting that cluster-based analysis is required to evaluate phenotypic shifts more accurately.

Moreover, a population of atypical M2-like macrophages producing proinflammatory cytokines appeared to accumulate during aging [81]. These special M2-like macrophages likely formed because of cell-intrinsic changes, e.g., ER stress or mitochondrial dysfunction during aging [81]. In our study, less than 20% of LYVE1+ M2-like macrophages in old skeletal muscle showed elevated expression of mRNAs encoding proinflammatory proteins, such as S100A8, S100A9, and SPP1, compared to the young [94]. Further studies are required to fully characterize these macrophages in skeletal muscle.

## 8. Involvement of Macrophages in Age-Associated Impairment of Skeletal Muscle Function

Changes in macrophage phenotypes during aging contribute to the reduced repair and regenerative capacity in old skeletal muscle. However, whether macrophages directly contribute to skeletal muscle aging, especially the loss of muscle mass and strength, remains largely unknown. Here, we discuss the role of macrophages in injury repair and regeneration in old skeletal muscle, as well as their role in the functional decline of skeletal muscle during aging.

### 8.1. Macrophage Involvement in the Aging-Related Decline of the Repair Capacity

The process of repairing injured skeletal muscle takes longer in older humans and mice. This delay is strongly associated with a shift in macrophage polarization and a prolonged proinflammatory state during the repair process in old skeletal muscle [133]. In agreement, a proinflammatory cytokine SPP1 (Osteopontin) was highly expressed in skeletal muscle macrophages in old mice [94,134]. Upon injury, SPP1 protein was significantly elevated in the SC niche in old skeletal muscle, and it suppressed the myogenic capacity of SCs [134]. SPP1 neutralization improved the repair and regeneration of old skeletal muscle, consistent with the notion of delayed repair and regeneration due to the proinflammatory environment of old skeletal muscle [134].

Contrary to above observations, it was shown that fewer proinflammatory macrophages infiltrate old skeletal muscle during early recovery stages in models of skeletal muscle disuse atrophy or eccentric contraction injury in mice and humans [130,131], and the delivery of proinflammatory macrophages to muscle experiencing disuse atrophy promoted the recovery of muscle strength in aged mice [135]. Similarly, lower levels of the proinflammatory cytokine IFNγ were measured at early injury repair stages in old skeletal muscle compared to young, leading to thinner myofibers and elevated fibrosis [136]. Along these lines, a reduction in IFNγ-responsive macrophages in old skeletal muscle was linked to decreased SC activation and impaired muscle regeneration [136]. Thus, further studies are required to fully elucidate the role of proinflammatory macrophages during injury repair in old skeletal muscle, but findings thus far indicate that skewed macrophage polarization impairs repair and regeneration in old skeletal muscle. In addition, old skeletal muscle shows elevated fibrotic response during injury repair because of altered FAP function [50,125], and M2 macrophages may in part contribute to the fibrosis by increasing the production of proline, required for collagen biosynthesis by fibroblasts, through an M2-arginase cascade [55,56].

### 8.2. Macrophage Involvement in Muscle Function Decline during Aging

Although the function of macrophages in skeletal muscle aging is poorly known, a recent study found that macrophages play an important role in neuromuscular junction (NMJ) deterioration during aging. Skeletal muscle contracts voluntarily by motoneurons and the NMJ is the structure that converts the excitation signal to contraction force. In the NMJ, presynaptic nerve endings packed with acetylcholine (Ach) connect to postsynaptic endplates enriched with Ach receptors (AchR) [23,137]. During aging, there is marked deterioration of the NMJ structure and function, along with increased presynaptic nerve branching and reduced neurotransmitter vesicles [23,137,138], and thinner postsynaptic endplates with less AchRs, especially in type II myofibers [23,137,138]. These aging-related changes promote myofiber denervation, reduce fiber size, and increase hybrid fibers.

Endoneurial macrophages, the resident macrophages of peripheral nerves, significantly increased in deteriorating NMJs in old mouse skeletal muscle [139]. Accordingly, depletion of endoneurial macrophages by a selective CSF1R inhibitor significantly improved NMJ morphology and muscle strength in old mice [139]. Thus, macrophages play a critical role in skeletal muscle aging by affecting the NMJ, although the molecular mechanisms and macrophage subgroups responsible for NMJ degeneration remain to be determined.

A recent study showed that depletion of p16+ cells helped to maintain muscle mass and function in old male mice [140]. Notably, CD68+ macrophages, but not PAX7+ SCs, were significantly reduced in mice depleted of p16+ cells [140]. Macrophages express p16 and senescence-associated β-galactosidase (SA-βGal) even in non-senescent status [141], and therefore, non-senescent macrophages were likely reduced in this paradigm, contributing to the preservation of muscle mass and function in aging skeletal muscle. Furthermore, the number of SA-βGal+ cells increased significantly after muscle injury, and the application of senolytics resulted in a reduction of SA-βGal+ cells and improved muscle regeneration in old mice [142]. In this study, >90% of SA-βGal+ cells were found to be CD11b+, indicating that the majority of SA-βGal+ cells were macrophages [142]. Deeper studies of the function of macrophages in skeletal muscle aging are needed.

## 9. Perspectives—Possible Involvement of Macrophage Subgroups in Skeletal Muscle Aging

Encouraging recent work has uncovered new aspects of macrophage action in skeletal muscle homeostasis and aging. Here, we discuss the possible function of specific macrophage subgroups in skeletal muscle aging, especially with the goal of identifying new intervention targets for senescent cell removal and NMJ preservation.

### 9.1. “Super-Phagocytic” Macrophages May Help Senescent Cell Removal

Senescent cells have both positive and negative effects on homeostasis. Senescent cells accelerate wound healing, suppress tumor progression, and reduce fibrosis, but they also induce inflammation, damage tissues, and exacerbate aging-related degeneration [143,144,145]. In agreement with these pleiotropic actions, removal of senescent cells delayed the onset of aging phenotypes but increased the incidence of tumorigenesis [146,147,148]. Thus, while senescent cells are required for normal physiologic responses, excessive senescent cells can be detrimental, as shown in the aging process.

p21+ senescent myofibers and p16+ senescent SCs and FAPs accumulate in old skeletal muscle [20,37]. Suppressing p16 production restored regenerative function of geriatric SCs [37], and application of senolytics improved muscle function in old mice, suggesting that a suppression of senescence ameliorates aging-related deterioration of skeletal muscle [20].

Macrophages remove senescent cells by phagocytosis [149,150,151]. Skeletal muscle macrophages showed strong phagocytic capacity, but a subset of the novel LYVE1−/MHCII^low^ macrophage subgroup displayed a particularly strong phagocytic capacity [94]. This subset (Cl4, Figure 1A) accounted for ~4% of total macrophages in skeletal muscle, and expressed high levels of FcγRIV and CD36 (FcγRIV+/CD36+). Interestingly, FcγRIV+/CD36+ macrophages in cyclophosphamide-treated lymphoma model mice also showed a “super-phagocytic” capacity [98]. The function of FcγRIV+/CD36+ macrophages has not been characterized in any tissue yet. The potential capacity of this “super-phagocytic” macrophages to remove senescent cells deserves further study.

### 9.2. Are GPNMB+ Macrophages Senescent Macrophages in Skeletal Muscle?

Senescent macrophages may contribute to skeletal muscle aging, as other senescent cells do. GPNMB was recently identified as a transmembrane marker of senescence [101]. Targeted elimination of GPNMB+ cells by diphtheria toxin or vaccination attenuated age-associated pathologies and behavioral phenotypes in normally aging mice, and extended the lifespan of Hutchinson–Gilford progeroid mice [101].

Notably, GPNMB+ macrophages increased strikingly during aging in skeletal muscle, from ~2.5% in total macrophages in the young to ~13% in the old (Figure 1B) [94]. Enrichment of senescent and lipid metabolism/transport genes suggested that this cluster most likely represents senescent macrophages in skeletal muscle. The crosstalk between senescence and lipids in this macrophage cluster and whether targeted depletion of this cluster alleviates overall skeletal muscle aging warrant further study.

### 9.3. S100A8+/A9+ Macrophages May Promote Neuromuscular Junction Decline in Aging Skeletal Muscle

Loss of muscle mass and strength occurs in many pathological conditions, including aging-related Alzheimer’s Disease (AD), where sarcopenic muscle loss often precedes cognitive impairment [152,153]. Amyloid-beta precursor protein (APP) is expressed in NMJ in mouse skeletal muscle [154,155], amyloid beta peptide (Aβ) levels increase in skeletal muscle in AD patients [156], and the presynaptic area of NMJ is strikingly reduced in AD model mice [157]. These findings suggest that Aβ accumulation may occur in peripheral nerves in skeletal muscle in AD.

S100A8 and S100A9 form a heterodimer, which serves as a proinflammatory biomarker that increases in macrophages in many inflammatory diseases [110]. Notably, S100A9 was significantly elevated in microglia in AD patients and AD mouse models [158,159,160,161]. Furthermore, S100A9 promoted inflammation and amyloid fibril formation [162,163], and ablation of the *S100a9* gene in AD mouse models reduced inflammation and Aβ production in brain [160], suggesting that S100A9 and its partner S100A8 may promote neurodegeneration in AD brain.

S100A8+/A9+ macrophages increased significantly in old skeletal muscle [94] and may exacerbate the degeneration of NMJs by promoting inflammation or Aβ production, and thus contribute to the sarcopenic muscle loss seen in AD patients. This process may also occur in normally aging skeletal muscle, as macrophages surrounding NMJs were shown to contribute to NMJ deterioration [139], although future work is required to elucidate the function of S100A8+/A9+ macrophages in skeletal muscle aging.

## 10. Concluding Remarks

Resident macrophages are a critical constituent cell population in skeletal muscle. Skeletal muscle macrophages are derived from both embryonic progenitor cells and adult bone marrow hematopoietic stem cells. Macrophages interact with SCs, FAPs, and other immune cells for skeletal muscle repair and regeneration, in turn polarizing dynamically in skeletal muscle to provide a remarkable functional heterogeneity. During aging, macrophage polarization is altered, causing diminished repair and regeneration that can lead to sarcopenia. Overall, macrophages show more proinflammatory traits in old skeletal muscle, but subgroup-based in-depth studies are needed to fully understand their function in inflammation, NMJ maintenance, senescence, and senescent cell removal during skeletal muscle aging and pathogenic skeletal muscle diseases.
